# Successful anesthetic management of separation surgery for pygopagus conjoined twins: a case report

**DOI:** 10.1186/s40981-020-00406-8

**Published:** 2021-01-06

**Authors:** Yukari Sato, Akira Iura, Yu Kawamoto, Shunsuke Yamamoto, Takeshi Iritakenishi, Yuji Fujino

**Affiliations:** 1 Department of Anesthesiology and Intensive Care Medicine, Osaka University Graduate School of Medicine, 2-2 Yamada-oka, Suita, Osaka, 565-0871 Japan; 2 Department of Anesthesiology, Jikei University School of Medicine, 3-19-18 Nishi-shimbashi, Minato-ku, Tokyo, 105-8471 Japan

**Keywords:** Conjoined twins, Separation surgery, Position change

## Abstract

**Background:**

Conjoined twins are an extremely rare congenital occurrence, and anesthetic management for surgical separation presents unique challenges for anesthesiologists.

**Case presentation:**

Five-month-old male pygopagus conjoined twins underwent separation surgery. We performed anesthesia induction in the supine position and surgery in the prone position. This presented a challenge because the transition from supine to prone position reversed the positional relationship between the two babies, resulting in crossing of the respiratory circuits and monitors. To solve the problem, we used anesthesia machines and monitors on the opposite side of each baby during anesthesia induction. The positional relationship between the twins and anesthesia machines and monitors normalized after the change to the prone position. Following the separation surgery, the twins were discharged without any complications.

**Conclusions:**

Our method of using opposite side anesthetic machines and monitors for anesthesia induction was useful for the safe anesthetic management of pygopagus conjoined twins.

## Background

The incidence of conjoined twins is extremely rare, ranging from 1/50,000 to 1/200,000 live births [1]. The anesthetic management of conjoined twins undergoing separation surgery is challenging and complex, requiring cooperation with the surgical team. A previous study emphasized the importance of preoperative examination and a multidisciplinary approach to surgical planning in these cases [2].

Herein, we describe in detail the successful anesthetic management in a surgery to separate pygopagus conjoined twins. In this case, the relationship between the left and right babies was reversed by a change in position from supine to prone, which presented unique challenges. The twins’ parents provided written consent for the publication of this report.

## Case presentation

**Fig. 1:**
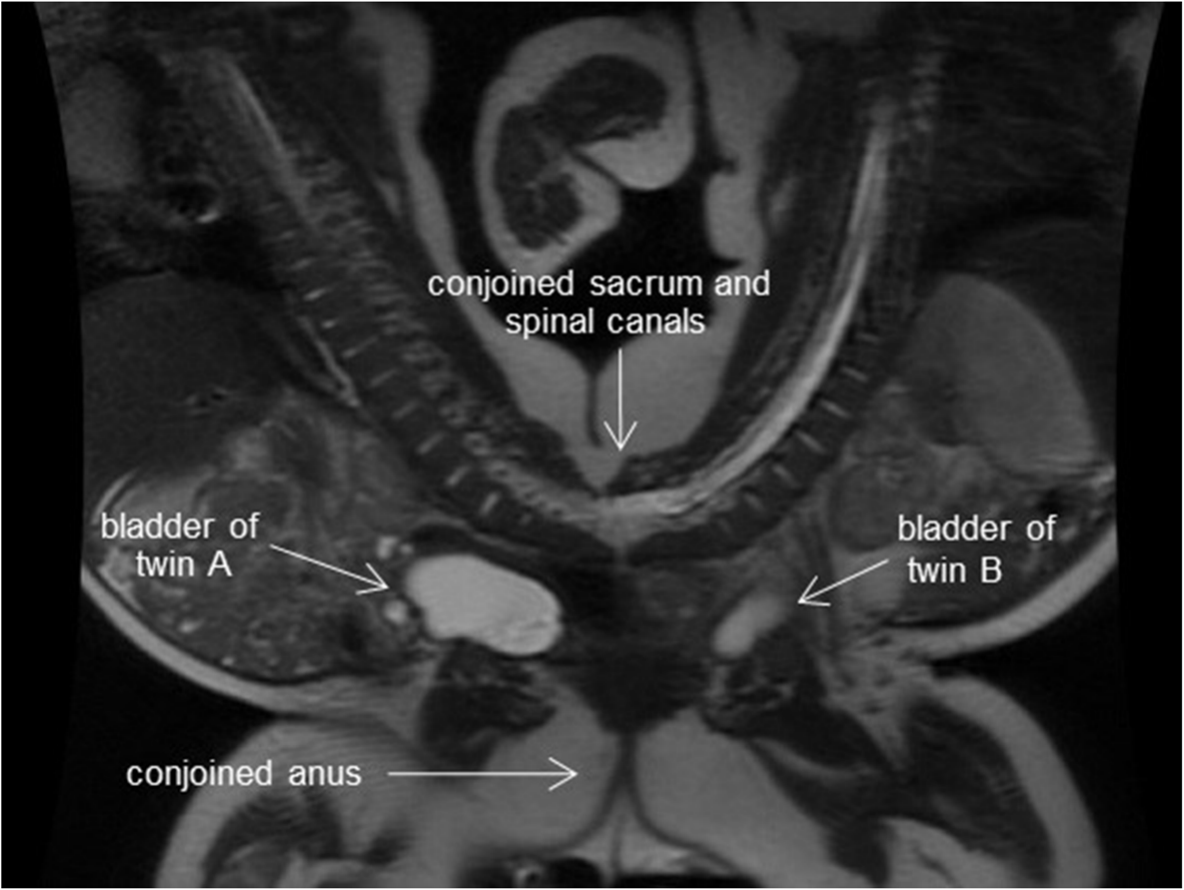
Magnetic resonance imaging of the pygopagus twins; they shared the sacrum, coccyx, anus, and urethra. Each had a separate bladder

Suspected pygopagus conjoined twin boys (prenatal diagnosis) were born by elective cesarean section at 35 weeks gestation. Their total birth weight was 4.69 kg; they did not require resuscitation, and their Apgar scores at 1 and 5 min after birth were 8 and 9, respectively, in both twins. Magnetic resonance imaging revealed that they were conjoined from the sacrum to the perineum, sharing the sacrum, coccyx, anus, and urethra (Fig. 1). Neither baby had any other apparent abnormalities. Their growth after birth was satisfactory, and the separation surgery was scheduled at 5 months. The total weight of the twins at the time of surgery was 13.8 kg, with both twins being approximately the same size. Conjoined twins share major/minor blood vessels, resulting in varying degrees of cross-circulation. The imaging results suggested that cross-circulation was limited in this case.

The multidisciplinary team comprised specialists in pediatric surgery, urology, neurosurgery, plastic surgery, anesthesiology, and operating room nursing. The team conducted two simulations in the operating room. The first simulation was conducted a month before the surgery to check the order of the surgery and equipment layout. The second simulation included the twins and was conducted 1 week before the actual surgery to check the surgical position. The surgery was performed in the largest operating room available in the hospital. The room was equipped with two operating tables, two anesthesia machines, and two patient monitors. The twins were managed by two anesthesia teams, with each team comprising one anesthetic consultant and one trainee.

On the day before the operation, separate peripheral intravenous lines and double-lumen peripherally inserted central catheters were secured on each baby. No premedication was administered. Standard American Society of Anesthesiologists monitors were attached to each baby. Propofol (10 mg), fentanyl (10 μg), and rocuronium (5 mg) were injected, and tracheal intubation was performed in twin A first. During the intubation sequence in twin A, twin B showed no sedation and remained awake. The same anesthesia induction sequence was subsequently performed in twin B. Additional peripheral intravenous and arterial lines were secured in both babies. Anesthesia was maintained with sevoflurane and remifentanil.

**Fig. 2:**
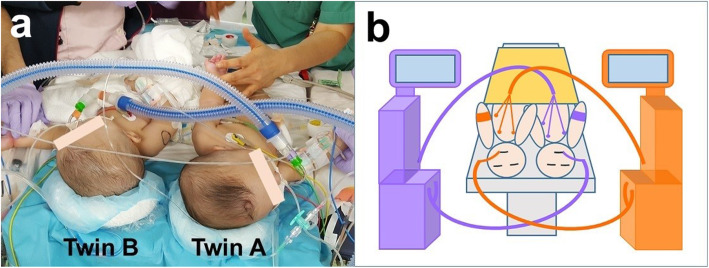
**a** The twins in supine position. Twin A and twin B were labeled purple and orange, respectively. **b** Schematic diagram in supine position. Anesthetic circuits and monitors crossed while the twins were in the supine position

**Fig. 3:**
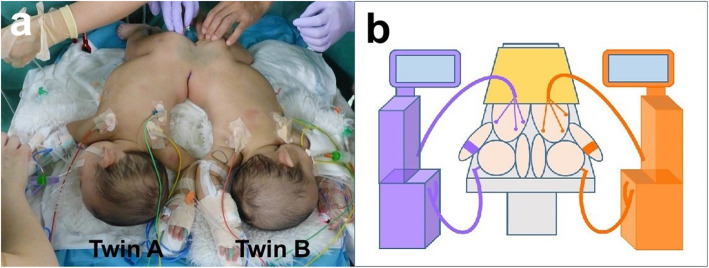
**a** The twins in prone position. **b** Schematic diagram in prone position. Anesthetic circuits and monitors normalized after the twins were changed to the prone position

The twins were positioned supine (Fig. 2a) at the beginning of surgery. After cystoscopy and urinary catheter placement, the twins were changed to the prone position (Fig. 3a). Initially, the sacrum, coccyx, and cauda equine were separated, followed by the common rectum. Bladder and ureter separation were performed last. Although their spinal canals were connected, the spinal cords were not fused. The neurosurgeon used nerve integrity monitoring during caudal equine separation. Hence, we avoided the use of muscle relaxant during the monitoring. The twins were separated approximately 10 h after the initiation of surgery. Twin A was moved to a vacant operating table in the same room where two surgical teams performed construction of the urethra and anus. Arterial blood gas analyses for each baby were performed approximately every 2 h. The total surgery time was approximately 15 h, with a total intraoperative blood loss of 30 mL. The administered intraoperative fluids and blood products comprised 568 mL and 350 mL of packed red blood cells, 750 mL and 700 mL of albumin products, and 1700 mL and 1550 mL of crystalloid solution for twin A and twin B, respectively. Neither hypothermia nor hypotension was observed intraoperatively in either baby. They were transferred to the intensive care unit for mechanical ventilation and were extubated on postoperative day 2. Their postoperative course was uneventful, and they were discharged on postoperative day 35 without any complications. Both twins had no developmental problem after surgery and were able to walk at 14 months of age.

## Discussion

Separation surgery for conjoined twins is extremely long and entails massive blood loss and fluid shifts; therefore, it should not be performed during the neonatal period [3]. To facilitate safer management, it is usually planned for between the postnatal age of 4 and 11 months [4]. In the present case, the general health of both twins was stable and it was decided that the surgery would be performed at 5 months after birth.

Conjoined twins are classified according to the site of conjunction: chest (thoracopagus), abdomen (omphalopagus), sacrum (pygopagus), pelvis (ischiopagus), and head (craniopagus). Thoracopagus conjunction is the most common type and is associated with a significant risk for respiratory and cardiovascular complications. Some omphalopagus twins have fused livers in which case blood loss may be considerable during separation surgery. Airway management can be challenging due to positioning issues in thoracopagus and omphalopagus [1].

The current case was pygopagus, which accounts for approximately 6–19% of all conjoined twins [4, 5]. Because of the rotation of the spine, the upper bodies of both twins were in a supine position and they could be positioned side by side. They had no facial abnormalities and difficult mask ventilation was not anticipated. The effects of cross-circulation should be assessed, especially in thoracopagus and omphalopagus twins. In the current pygopagus case, preoperative imaging studies revealed limited shared circulation. Based on these respiratory and circulatory evaluations, intravenous induction was planned.

The anesthetic management of conjoined twins is challenging and requires cooperation between a large number of medical staff. A preoperative examination is essential, including the site of attachment, shared organ systems, any complications that are present, and airway assessment. Multidisciplinary team preparation is key to the successful management of separation surgery [4, 6]. It is also recommended that simulations be performed in the operating room [2, 7]. The team conducted surgical planning and simulations twice and consequently established good communication.

Two anesthesia machines, two anesthesia medication carts, and two patient monitors were used in one large operating theater. To avoid any confusion, color-coding is recommended [4, 8]. All infusion lines, equipment, respiratory systems, and drugs were color-coded for each twin (i.e*.*, purple for twin A and orange for twin B; see Figs. 2b and Fig. 3 b).

The main intraoperative complication was the positional change of the babies. Prone position was required during the surgery for anatomical reasons. In the present case, the relationship between the left and right babies was reversed by a position change from supine to prone, causing the monitors and respiratory circuits to cross over. We realized the problem during the second simulation. Changes in the position of the babies also posed a risk for medication-related errors and dislocation of endotracheal tubes [9]. A drug administration error resulting from the crossover of venous lines in the prone position has been reported in a similar case [6]. To reduce this risk, we used the anesthesia machine and monitor on the opposite side during anesthesia induction in both twins. That is, at the time of anesthesia induction, the anesthesia machine and monitor on the right side were used for the baby on the left side and the equipment on the left side was used for the baby on the right side (Fig. 2b). The positional relationship between the babies and the anesthesia machines and monitors normalized after they were changed to the prone position (Fig. 3b).

In conclusion, anesthesiologists should be aware of the intersection of respiratory circuits and arteriovenous lines due to the change of position from supine to prone in separation surgery for conjoined twins. We used the anesthesia machines and monitors on the opposite side of each twin during anesthesia induction. This method was effective for distinguishing the twins, avoiding drug administration errors, and preventing associated problems and confusion throughout the operation.

## Data Availability

Data relevant to this case report are unavailable for public access because of patient privacy concerns.
